# Diagnostic Performance of Diffusion-Weighted Imaging for Differentiating Malignant From Benign Intraductal Papillary Mucinous Neoplasms of the Pancreas: A Systematic Review and Meta-Analysis

**DOI:** 10.3389/fonc.2021.637681

**Published:** 2021-07-05

**Authors:** Fan Xu, Yingying Liang, Wei Guo, Zhiping Liang, Liqi Li, Yuchao Xiong, Guoxi Ye, Xuwen Zeng

**Affiliations:** ^1^ Department of Radiology, Guangzhou Red Cross Hospital, Medical College, Jinan University, Guangzhou, China; ^2^ Department of Radiology, Guangzhou First People’s Hospital, School of Medicine, South China University of Technology, Guangzhou, China; ^3^ Department of Radiology, Wuhan Third Hospital (Tongren Hospital of WuHan University), Wuhan, China

**Keywords:** intraductal papillary mucinous neoplasms, DWI, diagnosis, meta-analysis, pancreas

## Abstract

**Objectives:**

To assess the diagnostic accuracy of diffusion-weighted imaging (DWI) in predicting the malignant potential in patients with intraductal papillary mucinous neoplasms (IPMNs) of the pancreas.

**Methods:**

A systematic search of articles investigating the diagnostic performance of DWI for prediction of malignant potential in IPMNs was conducted from PubMed, Embase, and Web of Science from January 1997 to 10 February 2020. QUADAS-2 tool was used to evaluate the study quality. Pooled sensitivity, specificity, diagnostic odds ratio (DOR), positive likelihood ratios (PLR), negative likelihood ratios (NLR), and their 95% confidence intervals (CIs) were calculated. The summary receiver operating characteristic (SROC) curve was then plotted, and meta-regression was also performed to explore the heterogeneity.

**Results:**

Five articles with 307 patients were included. The pooled sensitivity and specificity of DWI were 0.74 (95% CI: 0.65, 0.82) and 0.94 (95% CI: 0.78, 0.99), in evaluating the malignant potential of IPMNs. The PLR was 13.5 (95% CI: 3.1, 58.7), the NLR was 0.27 (95% CI: 0.20, 0.37), and DOR was 50.0 (95% CI: 11.0, 224.0). The area under the curve (AUC) of SROC curve was 0.84 (95% CI: 0.80, 0.87). The meta-regression showed that the slice thickness of DWI (p = 0.02) and DWI parameter (p= 0.01) were significant factors affecting the heterogeneity.

**Conclusions:**

DWI is an effective modality for the differential diagnosis between benign and malignant IPMNs. The slice thickness of DWI and DWI parameter were the main factors influencing diagnostic specificity.

## Introduction

Intraductal papillary mucinous neoplasms (IPMNs) of the pancreas, originating from the mucinous epithelium of the pancreatic duct system, are the most common types of pancreatic cystic neoplasms, which could overproduce mucin and lead to duct dilation (1, 2). Histologically, IPMNs are classified as low-grade, intermediate-grade, high-grade, or even invasive carcinoma depending on the variable degree of dysplasia, and these types have been found to be associated with different prognosis (3). Due to the variable risk of malignancy ranging from 6 to 40% (4, 5), it is crucial to accurately predict the malignant potential of IPMNs in order to choose appropriate surveillance and management strategy based on malignancy risk.

Diffusion-weighted imaging (DWI) is a functional MRI technique that reflects Brownian motion of free water and provides a qualitative or quantitative measurement of the motion of water molecules in various diseases by measuring the apparent diffusion coefficient (ADC) using the monoexponential model (6–9). Many studies have proved that high grade or invasive IPMNs demonstrate significantly lower ADC values than low- or moderate-grade IPMNs (10–12). Although a recent meta by Liu (13) found that MRI/MRCP had the highest pooled diagnostic accuracy and DWI had the highest pooled specificity in distinguishing benign and malignant IPMNs, few details about the DWI has been described in their study.

Therefore, the purpose of this study was to systematically evaluate the diagnostic performance of DWI for predicting the malignant potential of pancreatic IPMNs using a meta-analysis.

## Materials and Methods

### Literature Search

We performed a comprehensive literature search in PubMed, Embase, and Web of Science to select original studies focusing on evaluating the accuracy of DWI in predicting the malignant potential of pancreatic IPMNs from January 1997 to February 10, 2020 according to the Preferred Reporting Items for Systematic reviews and Meta-Analyses (PRISMA) guidelines (14). The literature search terms were used as follows: (1) “Diffusion weighted imaging” or “diffusion-weighted” or “diffusion weighted MR” or “diffusion-weighted magnetic resonance imaging” or “DWI” or “apparent diffusion coefficient” or “ADC”; and (2) “pancreatic cyst” or “pancreatic cystic neoplasm” or “pancreatic cystic tumors” or “intraductal papillary mucinous neoplasm” or “IPMN”. In addition, all the references of the included the study were checked and screened to ensure a comprehensive search. Two reviewers (FX and YL, with 5 and 7 years of experience) screened the literature independently, any discrepancies were resolved by discussion.

### Inclusion and Exclusion Criteria

The retrieved articles were first screened according to their titles and abstracts, and then full-text of potentially eligible articles were reviewed by the previously noted two reviewers independently. Any discrepancies were resolved by discussion.

The inclusion criteria were as follows: 1) original studies focused on evaluating the diagnostic performance of DWI in predicting the malignant potential of pancreatic IPMNs; 2) sufficient data to calculate the 2 × 2 table including the true positives (TPs), false positives (FPs), false negatives (FNs), and true negatives (TNs); 3) pathological results as the reference standard; and 4) articles published in English.

The exclusion criteria were as follows: 1) articles in the form of conference abstracts, reviews, case reports, editorials, letters, or animal studies; 2) studies not in the field of interest; and 3) studies with overlapping patients and data (the study with the largest study population was included).

### Data Extraction and Quality Assessment

The following data were extracted from the included studies: 1) study characteristics: publication years, authors, country, study period, study design, patient recruitment, blind, reader experience, patient numbers and ages, lesion numbers, reference standard, time interval between imaging test and surgery; 2) MRI techniques: vendor, scanner model, magnetic field strength, coil channels, DWI sequence, respiration, b values, slice thickness, diffusion restriction, and ADC cutoff values; 3) data was calculated for TPs, FPs, FNs, and TNs.

The quality assessment was evaluated using the Quality Assessment of Diagnostic Accuracy Studies-2 (QUADAS-2) tool (15). Data extraction and the quality assessment were performed by the previously noted two reviewers independently and disagreement was resolved at a consensus.

### Data Synthesis and Statistical Analysis

The forest plots of sensitivity and specificity were summarized in each study. The pooled sensitivity and specificity and their 95% confidence intervals (CIs) were obtained according to bivariate random-effects model. In addition, the positive likelihood ratio (PLR), negative likelihood ratio (NLR), and diagnostic odds ratio (DOR) with their 95% CIs were also obtained. Then, the summary receiver operating characteristic (SROC) curve was constructed, and area under the SROC curve (AUC) was computed to evaluate the value of DWI in diagnosing the malignant potential of IPMN, and the value was considered good for AUC value >0.9 and medium for AUC value from 0.7 to 0.9.

Heterogeneity among the studies was evaluated by Cochran’s Q-test (P < 0.05 indicating the presence of heterogeneity) and Higgins inconsistency index (I^2^) test [I^2^>50% indicating the presence of heterogeneity (16)]. The spearman correlation coefficient was calculated, and the presence of a threshold effect was indicated by a P-value less than 0.05.

A meta-regression was conducted to explain the effects of heterogeneity, with the following covariates being evaluated using a bivariate model: (1) study enrollment (consecutive *vs.* not available); (2) reader experience (available *vs.* not available); (3) reader number (n = 1 *vs.* n = 2); (4) magnetic strength (1.5T *vs.* 3T); (5) max value of b value (<1,000 *vs.* ≥ 1,000); (6) thickness of DWI (5 *vs.* 7mm); and (7) DWI parameter (quantitative DWI *vs.* qualitative DWI).

A Deeks’ funnel plot was performed to evaluate publication bias, with statistical significance being assessed by Deeks’ asymmetry test.

Data analyses were performed using the Midas modules in Stata 15.0 (StataCorp, College Station, TX, USA). A value of p < 0.05 was considered as indicating statistical significance.

## Results

### Literature Search

**Figure 1:**
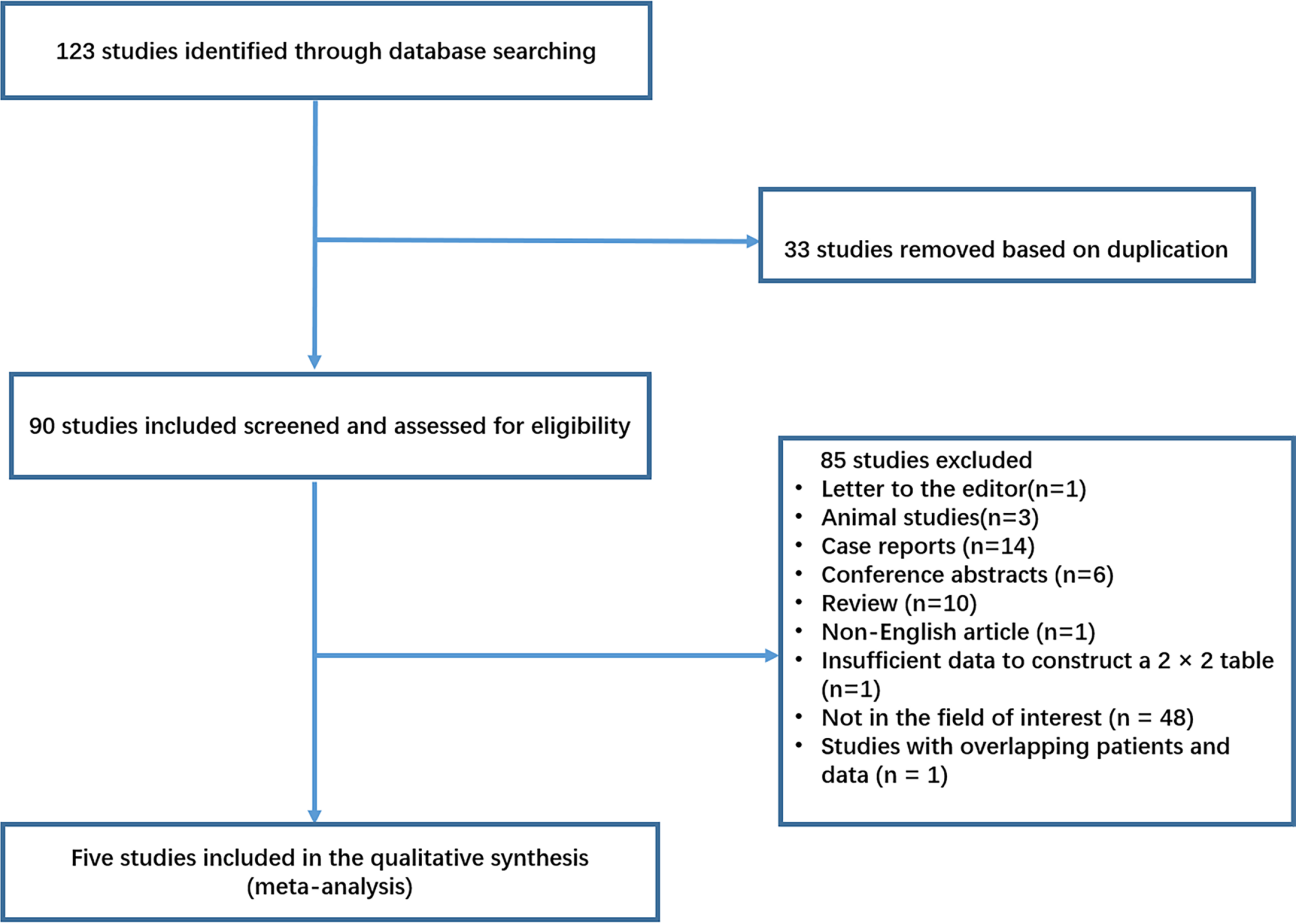
Flowchart showing selection process for meta-analysis.

**Figure 1** demonstrated a flowchart of the selection process. A total of 123 studies were identified according to the described search strategies, and 33 duplicate articles were removed. Subsequently, 85 studies were excluded for the following reasons: letter to the editor (n = 1), animal studies (n = 3), case reports (n = 14), conference abstract (n = 6), review (n = 10), non-English article (n = 1), insufficient data to construct a 2 × 2 table (n = 1), not in the field of interest (n = 48), or studies with overlapping patients and data (n = 1) (10) (**Figure 1**). Finally, five eligible articles with six studies were included in this meta-analysis.

### Characteristics of the Included Studies

The main study and MRI features were shown in **Tables 1** and **2**. All studies were retrospective. A total of 307 patients with 307 lesions were included ranging from 35 to 132. The number of malignant IPMNs (39.7%, 122/307) ranged from 15 to 49. All patients had histopathology after surgey as the reference standard. The 1.5T scanners were used in two studies (11, 17) and 3.0T scanners were used in three studies (18–20). The slice thickness of DWI was 5 mm in two articles (19, 20) and 7 mm in three articles (11, 17, 18). Max value of b value ≥1,000 was found in two articles (11, 18), and max value of b value<1,000 in three articles (17, 19, 20).

**Table 1:** Study characteristics of the included studies.

**First author**	**Year**	**Country**	**Study period**	**Design**	**Patient Recruitment**	**Blind**	**Reader(experience, years)**	**Age (years)**	**Patient number(M/F)**	**Lesion number(B**/**M)**	**Reference**	**Time from MR to reference**
											standard	
								mean (range)				
												standard (mean, range, days)
Zhang (17)	2016	China	2010.1-2015.6	R	NA	Yes	1 (10)	61.95 (NA)	42 (25/17)	42 (25/17)	Histopathology	7 (2–12)
											(surgery)	
Kang (18)	2014	Korea	2010.6-2012.7	R	NA	Yes	1 (3)	NA	37 (NA)	37 (22/15)	Histopathology	10 (0–64)
											(surgery)	
Kim (19)	2017	Korea	2013.8-2015.6	R	NA	Yes	2 (16/6)	NA	132 (83/49)	132 (83/49)	Histopathology	14.5 (1–31)
											(surgery)	
Ogawa (11)	2014	Japan	2005.6-2013.1	R	NA	Yes	2 (NA)	68 (NA)	35 (23/12)	35 (13/22)	Histopathology	NA (NA)
											(surgery)	
Jang (20)	2014	Korea	2008.1-2013.7	R	Consecutive	Yes	2 (13/3)	25.6 (35–83)	61 (34/27)	61 (42/19)	Histopathology	30 (NA)
											(surgery)	

M/F, male/female; R, retrospective; B, benign; M, malignant; NA, not available.

**Table 2:** MRI characteristics of the included studies.

**First author**	**Scanner**		**Magnetic strength (T)**	**Coil**	**Technical parameters**				**Interpretation**	
	Vendor	Model			DWI sequence	Respiration	b values for ADC maps (s/mm^2^)	Slice thickness (mm)	Diffusion restriction (yes/no)	ADC cut-off value (× 10^-3^) (mm^2^/s)
Zhang	Siemens	Magnetom Avanto;	1.5	NA	single-shot, echo-planar sequence	NA	0; 500	7	NA	2.66
Kang	Siemens	Magnetom Avanto;	3.0	32-channel phased-array coil	single-shot escho-planar	Free-breathing	0, 25, 50, 75, 100, 150, 200, 500, 800, 1,000	7	NA	1.99
Kim	Philips	Intera Achieva	3.0	16-channel phased-array receiver coil	single-shot escho-planar	Respiratory-triggered	0, 100, 800	5	Yes	NA
Ogawa	Toshiba	EXCELART Vantage	1.5	NA	single-shot escho-planar	Respiratory-triggered	0, 1,000	7	Yes	2.71
Jang	Philips	Intera Achieva	3.0	16-channel phased-array (torso or cardiac) coil	single-shot escho-planar	Respiratory-triggered	0, 100, 800	5	Yes	NA

DWI, diffusion-weighted imaging; ADC, apparent diffusion coefficient; NA, not available.

### Study Quality of the Included Studies

**Figure 2:**
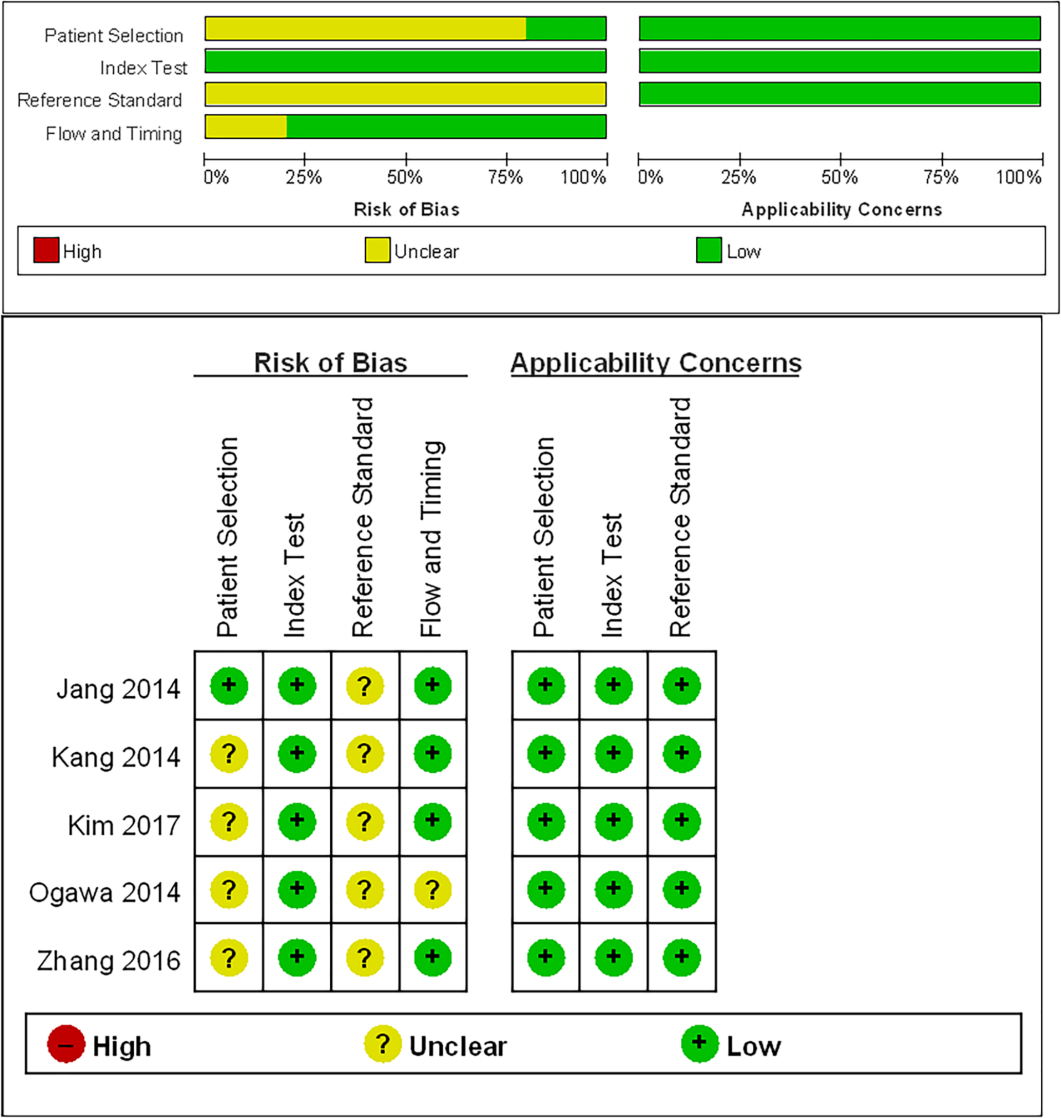
Quality Assessment of Diagnostic Accuracy Studies-2 (QUADAS-2) criteria for the five included studies.

The detailed study quality of the included studies was shown on **Figure 2**. Eighty percent studies (4/5) (11, 17–19) demonstrated unclear risk of bias in patient selection because they did not report the enrollment type of patient (consecutive or random). All five included studies were graded as an unclear risk of bias in reference standard because they did not describe whether the application of reference standard was blind. Twenty percent studies (1/5) (11) were graded as an unclear risk of bias in flow and timing due to the lack of information about the interval between MRI examination and reference standard.

### Overall Diagnostic Accuracy of DWI for Prediction of the Malignant Potential of Pancreatic IPMNs

**Figure 3:**
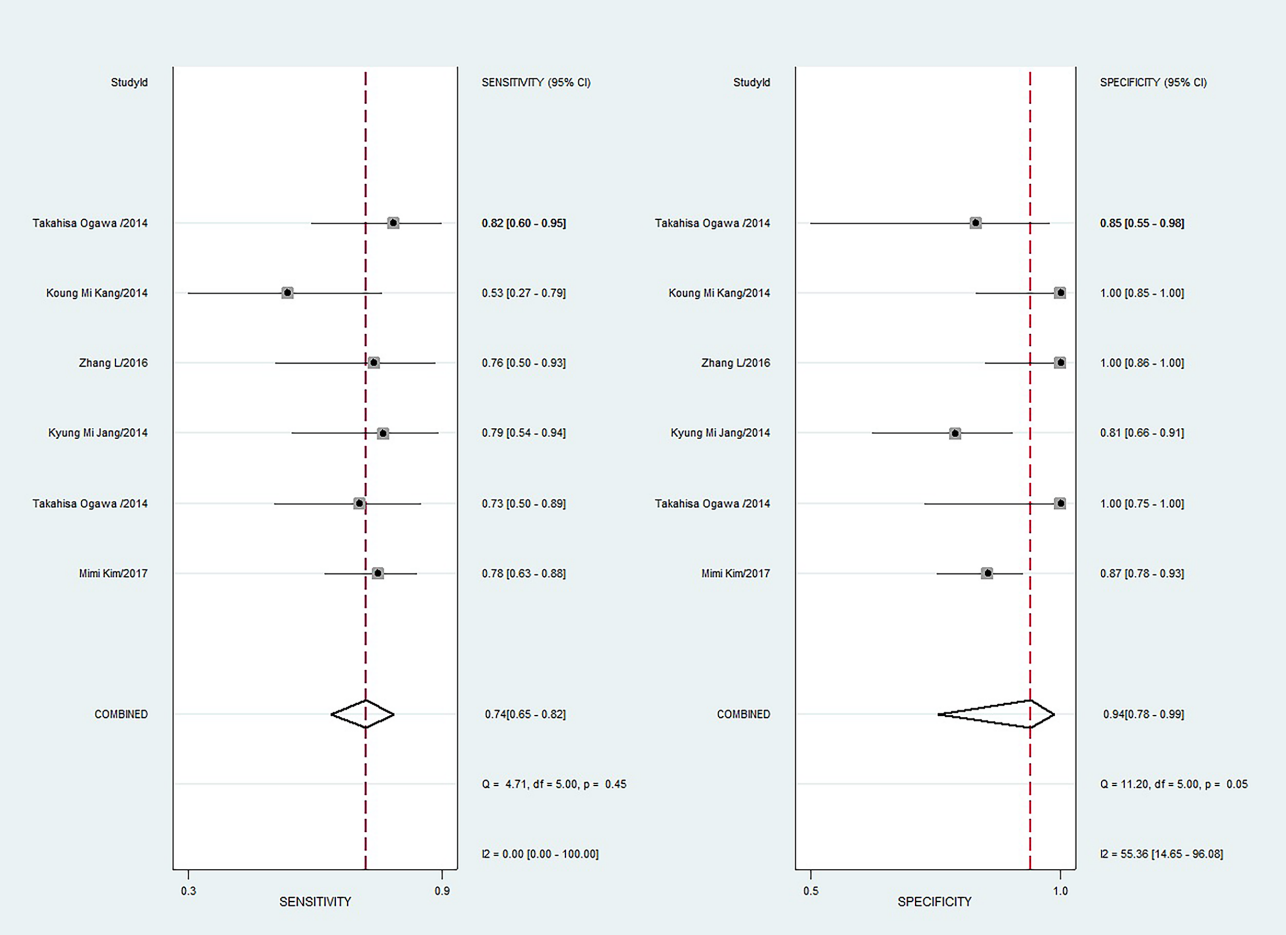
Coupled forest plots of pooled sensitivity and specificity. Numbers are pooled estimates with 95% confidence intervals (CIs) in parentheses and horizontal lines indicate 95% CIs.

**Figure 4:**
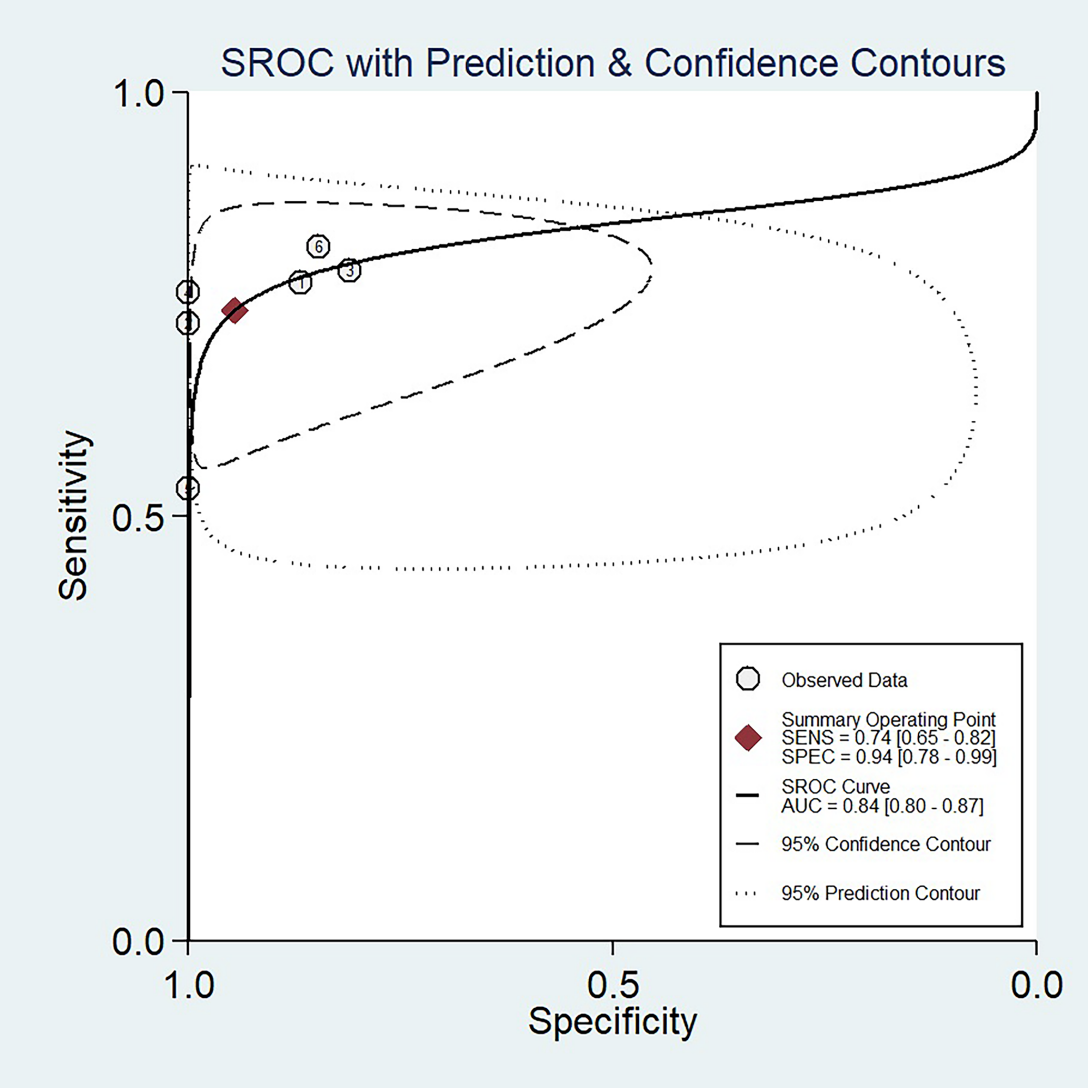
The summary receiver operating characteristic (SROC) curve of the diagnostic performance of DWI for differentiating malignant from benign IPMNs of the pancreas.

The sensitivity and specificity with 95% CIs of the six studies ranged from 0.53 to 0.82 and 0.81 to 1.00, respectively (**Figure 3**). The pooled sensitivity, specificity, PLR, NLR, and DOR for DWI in predicting malignant potential of pancreatic IPMNs were 0.74 (95% CI: 0.65, 0.82), 0.94 (95% CI: 0.78, 0.99), 13.5 (95% CI: 3.1, 58.7), 0.27 (95% CI: 0.20, 0.37), and 50.0 (95% CI: 11.0, 224.0), respectively. The AUC under the SROC curve was 0.84 (95% CI: 0.80, 0.87), which suggested medium diagnostic accuracy (**Figure 4**).

The Q test revealed no heterogeneity was present (Q = 3.948, p = 0.069). However, the Higgins I^2^ test demonstrated that heterogeneity was found in specificity (I^2^ = 55.36%, p = 0.05), not in sensitivity (I^2^ = 0, p = 0.45). The spearman correlation coefficient value of DWI was 0.771 (P = 0.072). This result showed that a threshold effect was absent in this meta-analysis.

**Figure 5:**
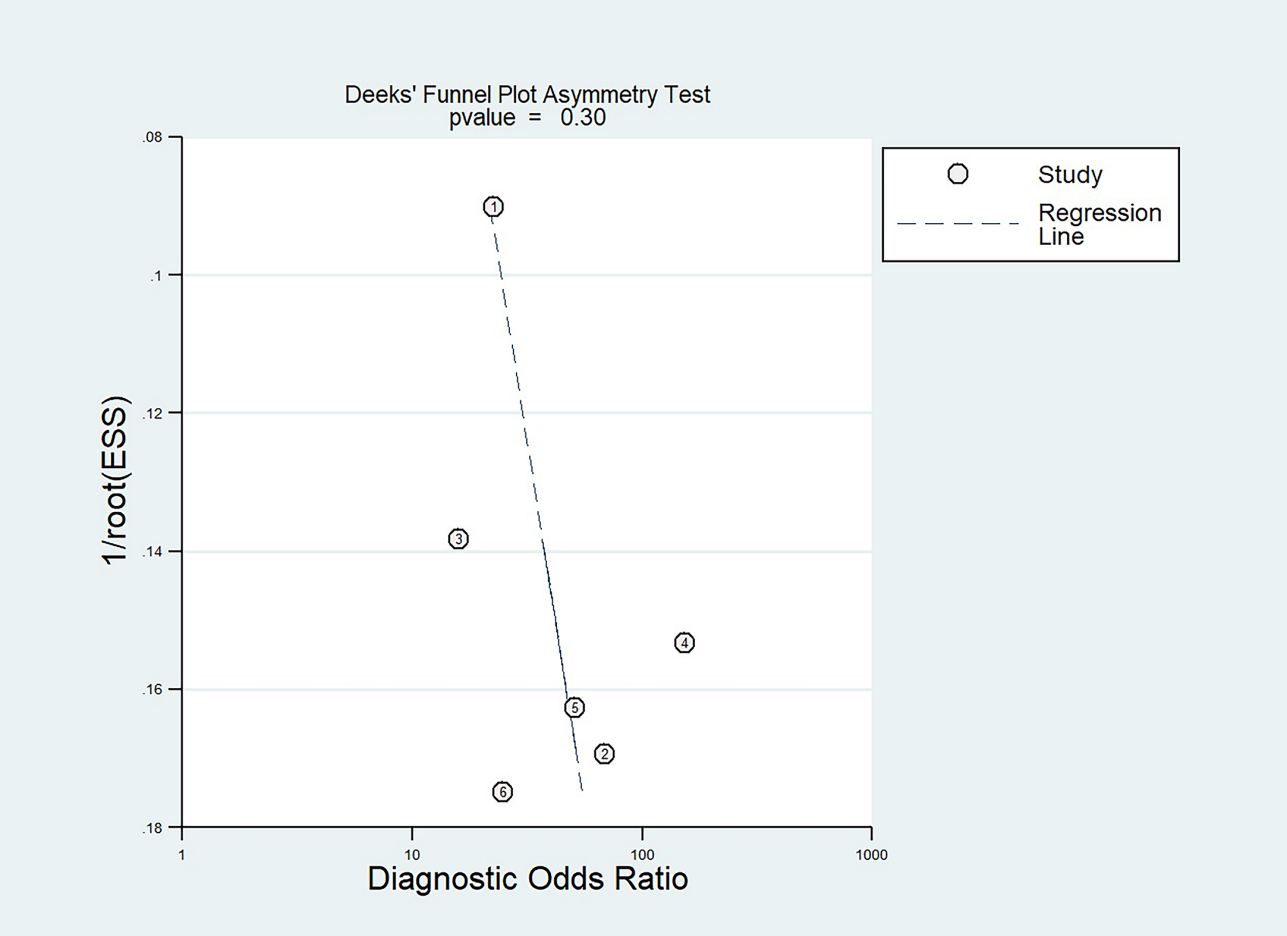
Deeks’ funnel plot used to evaluate potential publication bias.

The Deeks’ funnel plot and asymmetry test (P = 0.30 for the slope coefficient) both indicated no influence of publication bias on our meta-analysis (**Figure 5**).

### Meta-Regression Analyses

The results of the meta-regression analyses were summarized in **Table 3**. Among the variables that was considered a potential source of heterogeneity, the slice thickness of DWI (5 *vs.* 7 mm; p = 0.02) and DWI parameter (quantitative DWI *vs.* qualitative DWI; p = 0.01) were significant factors. Specifically, studies using a thickness = 5 mm showed a higher sensitivity (0.78 [95% CI 0.68–0.88]) compared with those using a thickness = 7 mm (0.72 [95% CI 0.62–0.82]); however, the pooled specificity estimates were not significantly different (0.85 [95% CI 0.79–0.91] *vs.* 0.97 [95% CI 0.94–1.00]; p = 0.19). Regarding the parameters of DWI, studies with quantitative DWI reported a significantly higher specificity (0.97 [95% CI 0.92–1.00] compared with those with qualitative DWI (0.86 [95% CI 0.80–0.92]); however, the pooled sensitivity estimates were not significantly different (0.72 [95% CI 0.60–0.84] *vs.* 0.77 [95% CI 0.68–0.85]); p = 0.21). Other factors, including the study enrollment, reader experience, number of readers, magnetic strength, and max value of b value did not significantly affect heterogeneity.

**Table 3:** Results of the meta-regression analysis of DWI for discriminating benign and malignant IPMN.

Covariates	Subgroup	Studies (n)	Meta-analytic summary estimates			
			Sensitivity	P	Specificity	p
					(95% CI)	
			(95% CI)			
Study enrollment	Consecutive enrollment	1	0.79 (0.59–0.99)	0.82	0.82 (0.54–1.00)	0.36
	NA	5	0.73 (0.64–0.82)		0.95 (0.88–1.00)	
Reader experience	Available	4	0.73 (0.62–0.83)	0.13	0.95 (0.85–1.00)	0.32
	NA	2	0.78 (0.63–0.92)		0.95 (0.83–1.00)	
Number of readers	n = 1	4	0.78 (0.70–0.85)	0.81	0.86 (0.81–0.92)	0.07
	n = 2	2	0.66 (0.49–0.82)		1.00 (1.00–1.00)	
Magnetic strength	1.5T	3	0.77 (0.65–0.90)	0.11	0.97 (0.91–1.00)	0.71
	3T	3	0.72 (0.60–0.85)		0.91 (0.79–1.00)	
Max value of b value	≥1,000	3	0.77 (0.67–0.87)	0.05	0.90 (0.78–1.00)	0.08
	<1,000	3	0.71 (0.58–0.84)		0.97 (0.91–1.00)	
Thickness of DWI	5 mm	2	0.78 (0.68–0.88)	0.02*	0.85 (0.79–0.91)	0.19
	7 mm	4	0.72 (0.62–0.82)		0.97 (0.94–1.00)	
DWI parameter	quantitative DWI	3	0.72 (0.60–0.84)	0.21	0.97 (0.92–1.00)	0.01*
	qualitative DWI	3	0.77 (0.68–0.85)		0.86 (0.80–0.92)	

*Denotes a statistical significance. DWI, diffusion-weighted imaging; NA, not available.

## Discussion

Our study demonstrated that DWI can accurately differentiate malignant potential of pancreatic IPMNs with overall pooled sensitivity of 74%, specificity of 94%, and AUC of 0.84. Heterogeneity was found in specificity (I^2^ = 55.36%), while not in sensitivity (I^2^ = 0). In meta-regression analyses, the DWI parameter and slice thickness of DWI were significant factors that affected the diagnostic performance of DWI in predicting malignant potential of IPMN.

The differential diagnosis of benign and malignant IPMNs is crucial for appropriate treatment and improving prognosis. Thus, it is imperative to identify an efficient and non-invasive mehod to detect malignant potential of IPMNs. DWI as a non-invasive imaging technique has been widely used in the pancreas disease (21–24). A recent meta by Liu et al. (13) also found that the pooled sensitivity, specificity, and AUC of DWI were 0.72, 0.97, and 0.82 in the differentiation of benign and malignant IPMNs, which was consisted with our results. MRCP is recommended for the diagnosis and follow-up of IPMN according to international guidelines. Kawakami et al. reported that the sensitivity and specificity of MRCP in differentiating malignant from benign IPMNs was only 60.5 and 93.9%, while the sensitivity and specificity of MRCP combined with DWI could be 92.1 and 91.2% (25). A study by Bertagna et al. (26) showed that F18-FDG-PET or PET/CT could achieve a better diagnosis performance between benign and malignant IPMNs with the pooled sensitivity and specificity of 88 and 98%, respectively. ADC entropy obtained from histogram analysis was also proved to be an effective predictive factor for identifying the malignant potential of IPMNs with comparable sensitivity (100 and 80%, respectively) and specificity (70 and 70.59%, respectively) (27, 28). Therefore, more prospective studies are required to confirm the diagnostic performance of DWI combined with many other advanced imaging techniques which may help and achieve the final diagnosis of IPMNs.

Many studies have proved that quantitative and qualitative DWI could be used to differentiate between malignant and benign tissues or assessing the tumor grade in various organs, including the lung (29, 30), liver (31), gallbladder (32), pancreas (10, 12, 17, 18), kidney (33, 34), and vertebral bone marrow (35, 36), because it could reflect microscopic motion of water protons at the cellular level (32, 33, 35, 37, 38). However, there may be some controversy in the performance of DWI on the diagnosis of IPMNs. Fatima et al. (39) reported that all IPMNs had low-iso signals on DWI without mention the malignancy of IPMNs, while other studies showed that the malignant IPMNs demonstrated a higher signal intensity and lower ADC value compared to benign IPMNs (10, 12, 17, 18).

Meta-regression analysis indicated that the slice thickness of DWI and DWI parameter were source of study heterogeneity. In particular, the pooled sensitivity was higher in studies with thinner slice thickness (5 mm) than those with thicker slice thickness (7 mm). This indicates that thinner slice thickness (5 mm) for the quantitative assessment of ADC is more appropriate for differentiating between benign and malignant IPMNs. You et al. (32) reported that qualitative DWI with sensitivity of 90% and specificity of 87% had a higher diagnostic performance compared to quantitative DWI with sensitivity of 82% and specificity of 86% in discriminating benign and malignant gallbladder lesions. However, it was reported by Shen et al. (29) that qualitative assessment and quantitative ADC could differentiate malignant from benign pulmonary lesions with reasonable accuracy (sensitivity: 0.88 *vs* 0.84; specificity: 0.75 *vs* 0.84) while they had no significant differences. Actually, our results indicated that the pooled specificity of quantitative DWI was more accurate than the qualitative DWI for differentiating benign and malignant IPMNs (0.97 *vs* 0.86, P = 0.01). This might be attributed to subjective assessment of qualitative DWI. Therefore, slice thickness of 5 mm and quantitative DWI were strongly recommended for DWI in differentiating benign and malignant IPMNs based on our results. However, due to the limited included studies, more further studies will be needed to confirm our results.

There were several limitations in this study. First, a relatively small number of the included studies without standard method of measuring ADC values was a major limitation, and this prevented calculation of diagnostic values in different patient subgroups. Second, there were only surgical series, and this probably underestimated the number of benign IPMNs. Third, it was still insufficient to explore the reasons for the heterogeneity using meta-regression because large heterogeneity was found between the studies. Finally, all the included studies were retrospective which may overestimate the diagnostic performance (40). Thus, further prospective studies are needed to confirm the diagnostic performance of DWI.

In conclusion, our study demonstrated that DWI had a considerable potential and value in the differential diagnosis of benign and malignant IPMNs. The slice thickness and parameter of DWI affected the diagnostic performance of DWI. More prospective studies are needed to validate the diagnostic value of DWI in the future.

## Data Availability Statement

The original contributions presented in the study are included in the article/supplementary material. Further inquiries can be directed to the corresponding author.

## Author Contributions

Conception and design: FX, YL, and WG. Acquisition of additional data: FX, YL, and WG. Analysis and interpretation of data: FX, YL, WG, ZL, LL, YX, and GY. Statistical analysis: FX, YL, and WG. Drafting of the manuscript: FX. Critical revision of the manuscript: FX, YL, WG, ZL, and XZ. Study supervision: XZ. All authors listed have contributed substantially to the design, data collection and analysis, and editing of the manuscript. All authors contributed to the article and approved the submitted version.

## Funding

This study has received funding by the Guangzhou Planned Project of Science and Technology (grant number: 202102010102) (XZ), Guangzhou Science and Technology Project of Health, China (grant number: 20211A010019) (FX), and Guangzhou Planned Project of Science and Technology (grant number: 202102010031) (YL).

## Conflict of Interest

The authors declare that the research was conducted in the absence of any commercial or financial relationships that could be construed as a potential conflict of interest.

## References

[1] Del Chiaro M Verbeke C Salvia R Kloppel G Werner J McKay C. European Experts Consensus Statement on Cystic Tumours of the Pancreas. Dig Liver Dis (2013) 45:703–11. 10.1016/j.dld.2013.01.010 23415799

[2] Tanaka M Fernandez-Del Castillo C Kamisawa T Jang JY Levy P Ohtsuka T. Revisions of International Consensus Fukuoka Guidelines for the Management of IPMN of the Pancreas. Pancreatology (2017) 17:738–53. 10.1016/j.pan.2017.07.007 28735806

[3] Katabi N Klimstra DS. Intraductal Papillary Mucinous Neoplasms of the Pancreas: Clinical and Pathological Features and Diagnostic Approach. J Clin Pathol (2008) 61:1303–13. 10.1136/jcp.2007.049361 18703569

[4] Xu MM Yin S Siddiqui AA Salem RR Schrope B Sethi A. Comparison of the Diagnostic Accuracy of Three Current Guidelines for the Evaluation of Asymptomatic Pancreatic Cystic Neoplasms. Med (Baltimore) (2017) 96:e7900. 10.1097/MD.0000000000007900 PMC5585501 28858107

[5] Hsiao CY Yang CY Wu JM Kuo TC Tien YW. Utility of the 2006 Sendai and 2012 Fukuoka Guidelines for the Management of Intraductal Papillary Mucinous Neoplasm of the Pancreas: A Single-Center Experience With 138 Surgically Treated Patients. Med (Baltimore) (2016) 95:e4922. 10.1097/MD.0000000000004922 PMC5044913 27661043

[6] Kwee TC Takahara T Ochiai R Katahira K Van Cauteren M Imai Y. Whole-Body Diffusion-Weighted Magnetic Resonance Imaging. Eur J Radiol (2009) 70:409–17. 10.1016/j.ejrad.2009.03.054 19403255

[7] Barral M Taouli B Guiu B Koh DM Luciani A Manfredi R. Diffusion-Weighted MR Imaging of the Pancreas: Current Status and Recommendations. Radiology (2015) 274:45–63. 10.1148/radiol.14130778 25531479

[8] Fukukura Y Shindo T Hakamada H Takumi K Umanodan T Nakajo M. Diffusion-Weighted MR Imaging of the Pancreas: Optimizing B-Value for Visualization of Pancreatic Adenocarcinoma. Eur Radiol (2016) 26:3419–27. 10.1007/s00330-015-4174-5 26738506

[9] Barral M Soyer P Ben Hassen W Gayat E Aout M Chiaradia M. Diffusion-Weighted MR Imaging of the Normal Pancreas: Reproducibility and Variations of Apparent Diffusion Coefficient Measurement at 1.5- and 3.0-Tesla. Diagn Interv Imaging (2013) 94:418–27. 10.1016/j.diii.2012.12.007 23415463

[10] Kang KM Lee JM Shin CI Baek JH Kim SH Yoon JH. Added Value of Diffusion-Weighted Imaging to MR Cholangiopancreatography With Unenhanced Mr Imaging for Predicting Malignancy or Invasiveness of Intraductal Papillary Mucinous Neoplasm of the Pancreas. J Magn Reson Imaging (2013) 38:555–63. 10.1002/jmri.24022 23390008

[11] Ogawa T Horaguchi J Fujita N Noda Y Kobayashi G Ito K. Diffusion-Weighted Magnetic Resonance Imaging for Evaluating the Histological Degree of Malignancy in Patients With Intraductal Papillary Mucinous Neoplasm. J Hepatobiliary Pancreat Sci (2014) 21:801–8. 10.1002/jhbp.135 25082473

[12] Sandrasegaran K Akisik FM Patel AA Rydberg M Cramer HM Agaram NP. Diffusion-Weighted Imaging in Characterization of Cystic Pancreatic Lesions. Clin Radiol (2011) 66:808–14. 10.1016/j.crad.2011.01.016 21601184

[13] Liu H Cui Y Shao J Shao Z Su F Li Y. The Diagnostic Role of CT, MRI/MRCP, PET/CT, EUS and DWI in the Differentiation of Benign and Malignant IPMN: A Meta-Analysis. Clin Imaging (2021) 72:183–93. 10.1016/j.clinimag.2020.11.018 33321460

[14] Hutton B Salanti G Caldwell DM Chaimani A Schmid CH Cameron C. The PRISMA Extension Statement for Reporting of Systematic Reviews Incorporating Network Meta-Analyses of Health Care Interventions: Checklist and Explanations. Ann Intern Med (2015) 162:777–84. 10.7326/M14-2385 26030634

[15] Whiting PF Rutjes AW Westwood ME Mallett S Deeks JJ Reitsma JB. QUADAS-2: A Revised Tool for the Quality Assessment of Diagnostic Accuracy Studies. Ann Intern Med (2011) 155:529–36. 10.7326/0003-4819-155-8-201110180-00009 22007046

[16] Higgins JP Thompson SG Deeks JJ Altman DG. Measuring Inconsistency in Meta-Analyses. BMJ (2003) 327:557–60. 10.1136/bmj.327.7414.557 PMC192859 12958120

[17] Zhang L Rao SX Xu XF Wang DS Jin da Y Zeng MS. Value of Apparent Diffusion Coefficient for Predicting Malignancy of Intraductal Papillary Mucinous Neoplasms of the Pancreas. Diagn Interv Radiol (2016) 22:308–13. 10.5152/dir.2016.15354 PMC4956014 27283593

[18] Kang KM Lee JM Yoon JH Kiefer B Han JK Choi BI. Intravoxel Incoherent Motion Diffusion-Weighted MR Imaging for Characterization of Focal Pancreatic Lesions. Radiology (2014) 270:444–53. 10.1148/radiol.13122712 24126370

[19] Kim M Mi Jang K Kim SH Doo Song K Jeong WK Kang TW. Diagnostic Accuracy of Diffusion Restriction in Intraductal Papillary Mucinous Neoplasm of the Pancreas in Comparison With “High-Risk Stigmata” of the 2012 International Consensus Guidelines for Prediction of the Malignancy and Invasiveness. Acta Radiol (2017) 58:1157–66. 10.1177/0284185116685921 28084815

[20] Jang KM Kim SH Min JH Lee SJ Kang TW Lim S. Value of Diffusion-Weighted MRI for Differentiating Malignant From Benign Intraductal Papillary Mucinous Neoplasms of the Pancreas. AJR Am J Roentgenol (2014) 203:992–1000. 10.2214/AJR.13.11980 25341136

[21] Kawakami S Fukasawa M Shimizu T Ichikawa S Sato T Takano S. Diffusion-Weighted Image Improves Detectability of Magnetic Resonance Cholangiopancreatography for Pancreatic Ductal Adenocarcinoma Concomitant With Intraductal Papillary Mucinous Neoplasm. Med (United States) (2019) 98:e18039. 10.1097/MD.0000000000018039 PMC6882617 31764824

[22] Pandey P Pandey A Shao NN Varzaneh FN Ghasaeh MA Zharghampour M. Added Value of Apparent Diffusion Coefficient in Distinguishing Between Serous and Mucin-Producing Pancreatic Cystic Neoplasms. Eur Radiol (2019) 29:4660–9. 10.1007/s00330-019-6010-9 30762111

[23] Hayashi M Mikata R Horikoshi T Senoo J Kusakabe Y Ohyama H. Diffusion-Weighted Magnetic Resonance Imaging and 18-Fluorodeoxglucose Positron Emission Tomography With Computed Tomography for Evaluating Malignancy of Branch Duct and Mixed Type Intraductal Papillary Mucinous Neoplasms of the Pancreas. Pancreas (2019) 48:E43–5. 10.1097/MPA.0000000000001316 31090668

[24] Kulali F Semiz-Oysu A Demir M Segmen–Yilmaz M Bukte Y. Role of Diffusion-Weighted MR Imaging in Predicting the Grade of Nonfunctional Pancreatic Neuroendocrine Tumors. Diagn Intervent Imaging (2018) 99:301–9. 10.1016/j.diii.2017.10.012 29258825

[25] Kawakami S Fukasawa M Shimizu T Ichikawa S Shimizu T Takano S. Diffusion-Weighted Image Improves Detectability of Magnetic Resonance Cholangiopancreatography for Pancreatic Ductal Adenocarcinoma Concomitant With Intraductal Papillary Mucinous Neoplasm. Med (Baltimore) (2019) 98:e18039. 10.1097/MD.0000000000018039 PMC6882617 31764824

[26] Bertagna F Treglia G Baiocchi GL Giubbini R. F18-FDG-PET/CT for Evaluation of Intraductal Papillary Mucinous Neoplasms (IPMN): A Review of the Literature. Jpn J Radiol (2013) 31:229–36. 10.1007/s11604-012-0176-2 23315020

[27] Jin KP Rao SX Sheng RF Zeng MS. Skewness of Apparent Diffusion Coefficient (ADC) Histogram Helps Predict the Invasive Potential of Intraductal Papillary Neoplasms of the Bile Ducts (IPNBs). Abdominal Radiol (2019) 44:95–103. 10.1007/s00261-018-1716-8 30151712

[28] Hoffman DH Ream JM Hajdu CH Rosenkrantz AB. Utility of Whole-Lesion ADC Histogram Metrics for Assessing the Malignant Potential of Pancreatic Intraductal Papillary Mucinous Neoplasms (IPMNs). Abdominal Radiol (2017) 42:1222–8. 10.1007/s00261-016-1001-7 27900458

[29] Shen G Ma H Liu B Ren P Kuang A. Diagnostic Performance of DWI With Multiple Parameters for Assessment and Characterization of Pulmonary Lesions: A Meta-Analysis. AJR Am J Roentgenol (2018) 210:58–67. 10.2214/AJR.17.18257 29091006

[30] Shen G Jia Z Deng H. Apparent Diffusion Coefficient Values of Diffusion-Weighted Imaging for Distinguishing Focal Pulmonary Lesions and Characterizing the Subtype of Lung Cancer: A Meta-Analysis. Eur Radiol (2016) 26:556–66. 10.1007/s00330-015-3840-y 26003791

[31] Wei C Tan J Xu L Juan L Zhang SW Wang L. Differential Diagnosis Between Hepatic Metastases and Benign Focal Lesions Using DWI With Parallel Acquisition Technique: A Meta-Analysis. Tumour Biol (2015) 36:983–90. 10.1007/s13277-014-2663-9 25318600

[32] You MW Yun SJ. Diagnostic Performance of Diffusion-Weighted Imaging for Differentiating Benign and Malignant Gallbladder Lesions: A Systematic Review and Meta-Analysis. J Magn Reson Imaging (2018) 48:1375–88. 10.1002/jmri.26035 29676860

[33] Woo S Suh CH Kim SY Cho JY Kim SH. Diagnostic Performance of DWI for Differentiating High- From Low-Grade Clear Cell Renal Cell Carcinoma: A Systematic Review and Meta-Analysis. AJR Am J Roentgenol (2017) 209:W374–w381. 10.2214/AJR.17.18283 29023154

[34] Li Y Wang Y Qin J Wu J Dai X Xu J. Meta-Analysis of Diffusion-Weighted Imaging in the Differential Diagnosis of Renal Lesions. Clin Imaging (2018) 52:264–72. 10.1016/j.clinimag.2018.08.010 30172176

[35] Suh CH Yun SJ Jin W Lee SH Park SY Ryu CW. ADC as a Useful Diagnostic Tool for Differentiating Benign and Malignant Vertebral Bone Marrow Lesions and Compression Fractures: A Systematic Review and Meta-Analysis. Eur Radiol (2018) 28:2890–902. 10.1007/s00330-018-5330-5 29450718

[36] Luo Z Litao L Gu S Luo X Li D Yu L. Standard-B-Value vs Low-B-Value DWI for Differentiation of Benign and Malignant Vertebral Fractures: A Meta-Analysis. Br J Radiol (2016) 89:20150384. 10.1259/bjr.20150384 26612466 PMC4985191

[37] Liang YY Xu F Guo Y Wang J. Diagnostic Accuracy of Magnetic Resonance Imaging Techniques for Parotid Tumors, a Systematic Review and Meta-Analysis. Clin Imaging (2018) 52:36–43. 10.1016/j.clinimag.2018.05.026 29908348

[38] Surov A Meyer HJ Wienke A. Apparent Diffusion Coefficient for Distinguishing Between Malignant and Benign Lesions in the Head and Neck Region: A Systematic Review and Meta-Analysis. Front Oncol (2019) 9:1362. 10.3389/fonc.2019.01362 31970081 PMC6960101

[39] Fatima Z Ichikawa T Motosugi U Muhi A Sano K Sou H. Magnetic Resonance Diffusion-Weighted Imaging in the Characterization of Pancreatic Mucinous Cystic Lesions. Clin Radiol (2011) 66:108–11. 10.1016/j.crad.2010.10.004 21216325

[40] Deeks JJ. Systematic Reviews in Health Care: Systematic Reviews of Evaluations of Diagnostic and Screening Tests. BMJ (2001) 323:157–62. 10.1136/bmj.323.7305.157 PMC1120791 11463691

